# Supplementary material for: Transfer learning efficiently maps bone marrow cell types from mouse to human using single-cell RNA sequencing
Source: Commun Biol. 2020 Dec 4;3:736. doi: 10.1038/s42003-020-01463-6 (PMC7718277; doi:10.1038/s42003-020-01463-6)
Supplement: Supplementary file 1 — Supplementary Information [file 42003_2020_1463_MOESM1_ESM.pdf]

Supplementary Information

related to

Transfer learning efficiently maps bone marrow cell types from mouse to human using single-cell RNA sequencing

Patrick S. Stumpf[1,2,*], Xin Du[3], Haruka Imanishi[4], Yuya Kunisaki[5], Yuichiro Semba[6], Timothy Noble[1], Rosanna C.G. Smith[7], Matthew Rose-Zerili[7], Jonathan J. West[7,8], Richard O.C. Oreffo[1,8], Katayoun Farrahi[3], Mahesan Niranjan[3], Koichi Akashi[6], Fumio Arai[4,*], Ben D. MacArthur[1,8,9,10,*]

1 Centre for Human Development, Stem Cells and Regeneration, Faculty of Medicine, University of Southampton, Southampton SO17 1BJ, UK
2 Joint Research Center for Computational Biomedicine, RWTH Aachen University, Aachen, 52074, Germany
3 Electronics and Computer Sciences, University of Southampton, Southampton, SO17 1BJ, UK
4 Kyushu University, Department of Stem Cell Biology and Medicine, Graduate School of Medical Sciences, Kyushu University, Fukuoka, 812-8582, Japan
5 Center for Cellular and Molecular Medicine, Kyushu University Hospital, Fukuoka, 812-8582, Japan
6 Department of Medicine and Biosystemic Science, Kyushu University Graduate School of Medical Sciences, Fukuoka, 812-8582, Japan
7 Cancer Sciences, Faculty of Medicine, University of Southampton, Southampton, SO16 6YD, UK
8 Institute for Life Sciences, University of Southampton, Southampton SO17 1BJ, UK
9 Mathematical Sciences, University of Southampton, Southampton, SO17 1BJ, UK
10 The Alan Turing Institute, London, NW1 2DB, UK
* Correspondence to: pstumpf@ukaachen.de, farai@med.kyushu-u.ac.jp, and bdm@soton.ac.uk

This document contains Supplementary Figures 1-8.

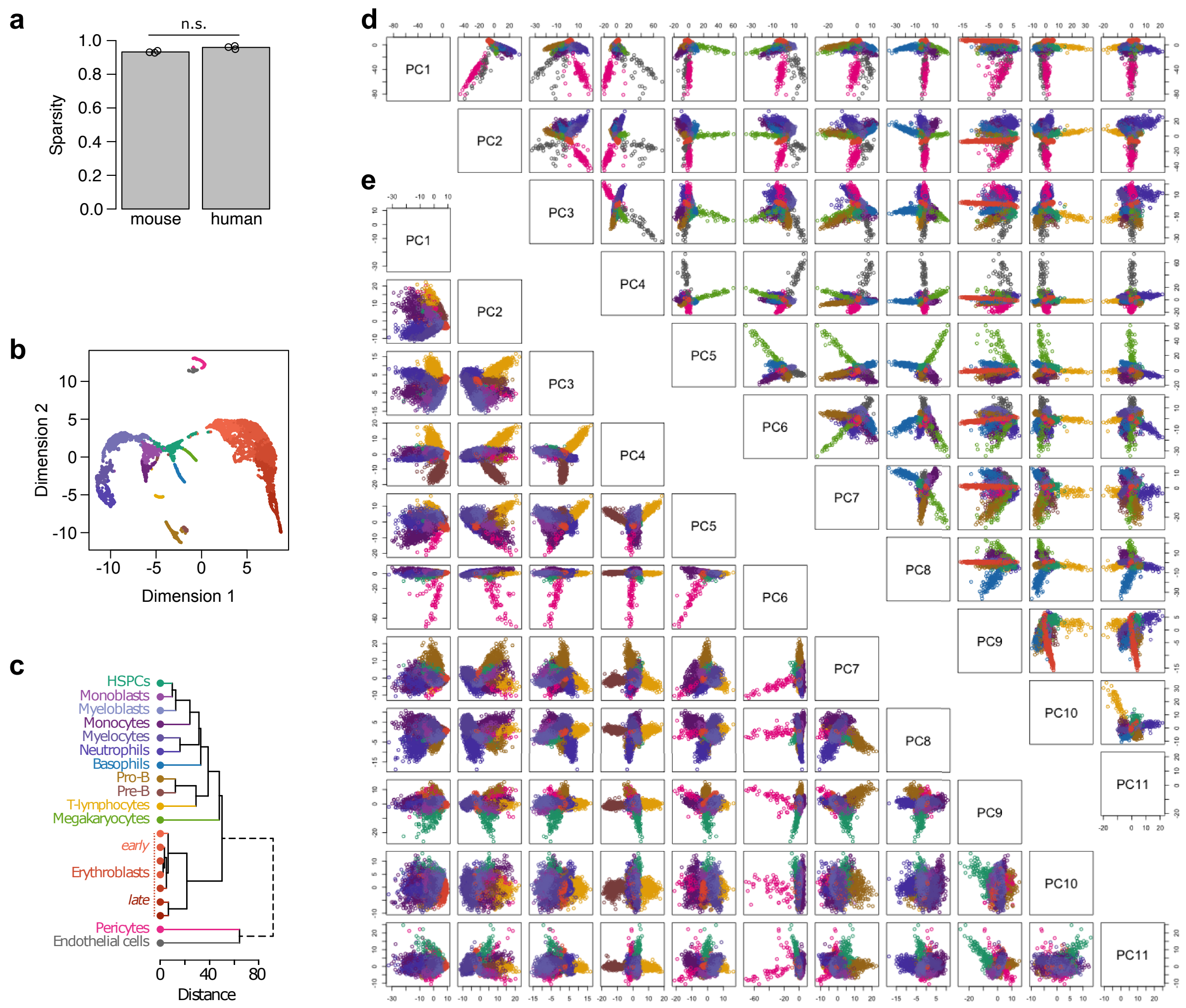

Supplementary Figure 1. Related to Fig. 1. a Sparsity (fraction of absent readings) in the single-cell data matrix. Circles indicate three biological replicates and bars indicate mean. The difference between human and mouse is not significant (p=0.1, Wilcoxon rank-sum test, n=3 independent biological samples). **b** Alternative projection of mouse bone marrow scRNAseq data onto two dimensions using UMAP. **c** Dendrogram of mouse bone marrow cell types using Euclidean distance between cluster median-centres, calculated from the first 11 principal components. **d** Principal component analysis of mouse data. **e** Principal component analysis of human data. Points in panels b,d, and e represent cells, coloured by annotation from unsupervised clustering as indicated in panel c.

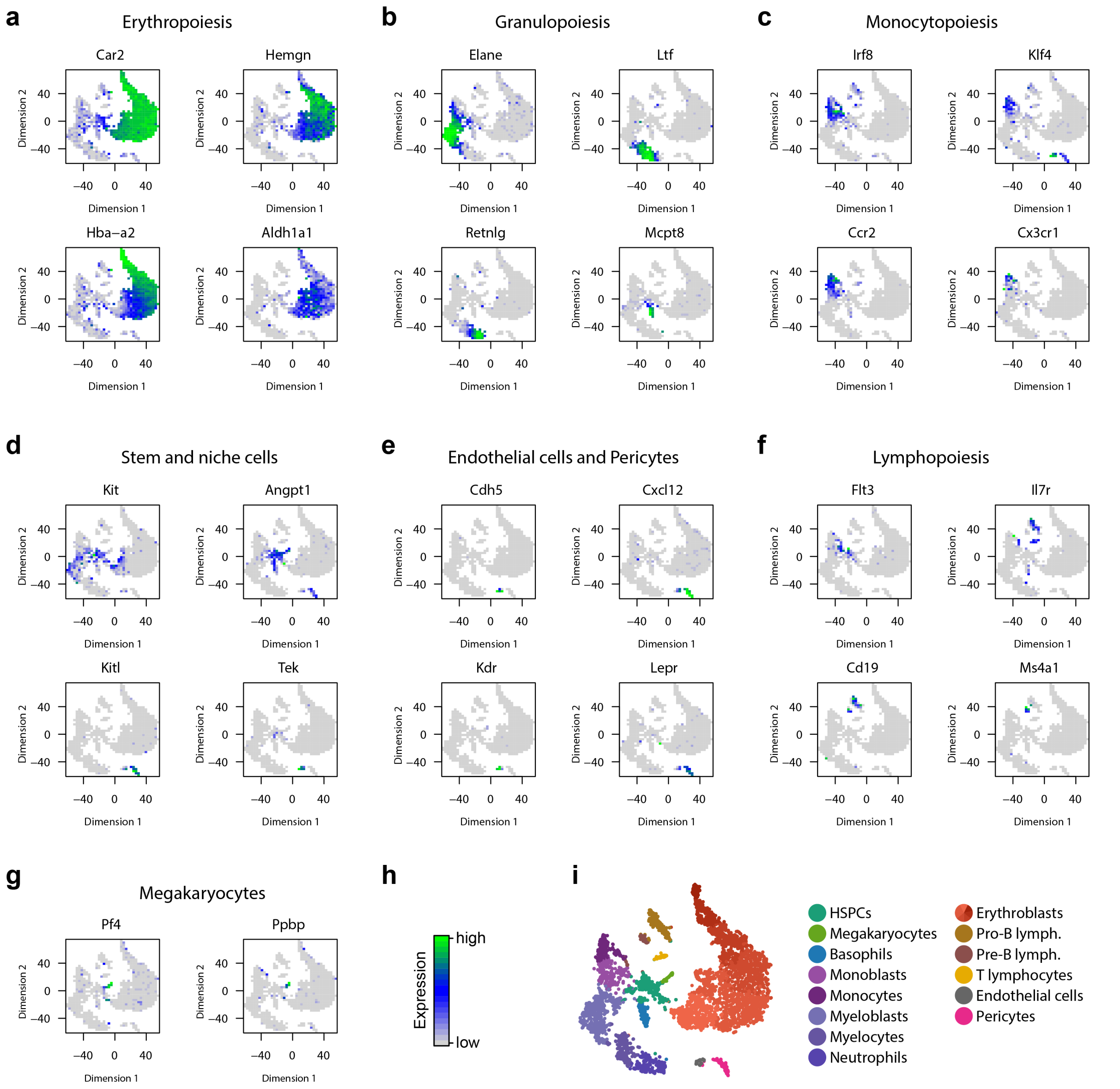

Supplementary Figure 2. Gene expression localizes to developmental branches of mouse hematopoiesis. a-g Average gene expression superimposed onto 2D embedding of scRNAseq data using tSNE. Displayed are the mean expression values for each 2D-bin. Localized expression indicative of **a** erythropoiesis, **b** granulopoiesis, **c** monocytopoiesis, **d** hematopoietic stem and progenitor cells and niche cells, **e** endothelial cells and pericytes, **f** lymphopoiesis, **g** thrombopoiesis. **h** Color scale. **i** Cluster structure from **Fig. 1c** for reference.

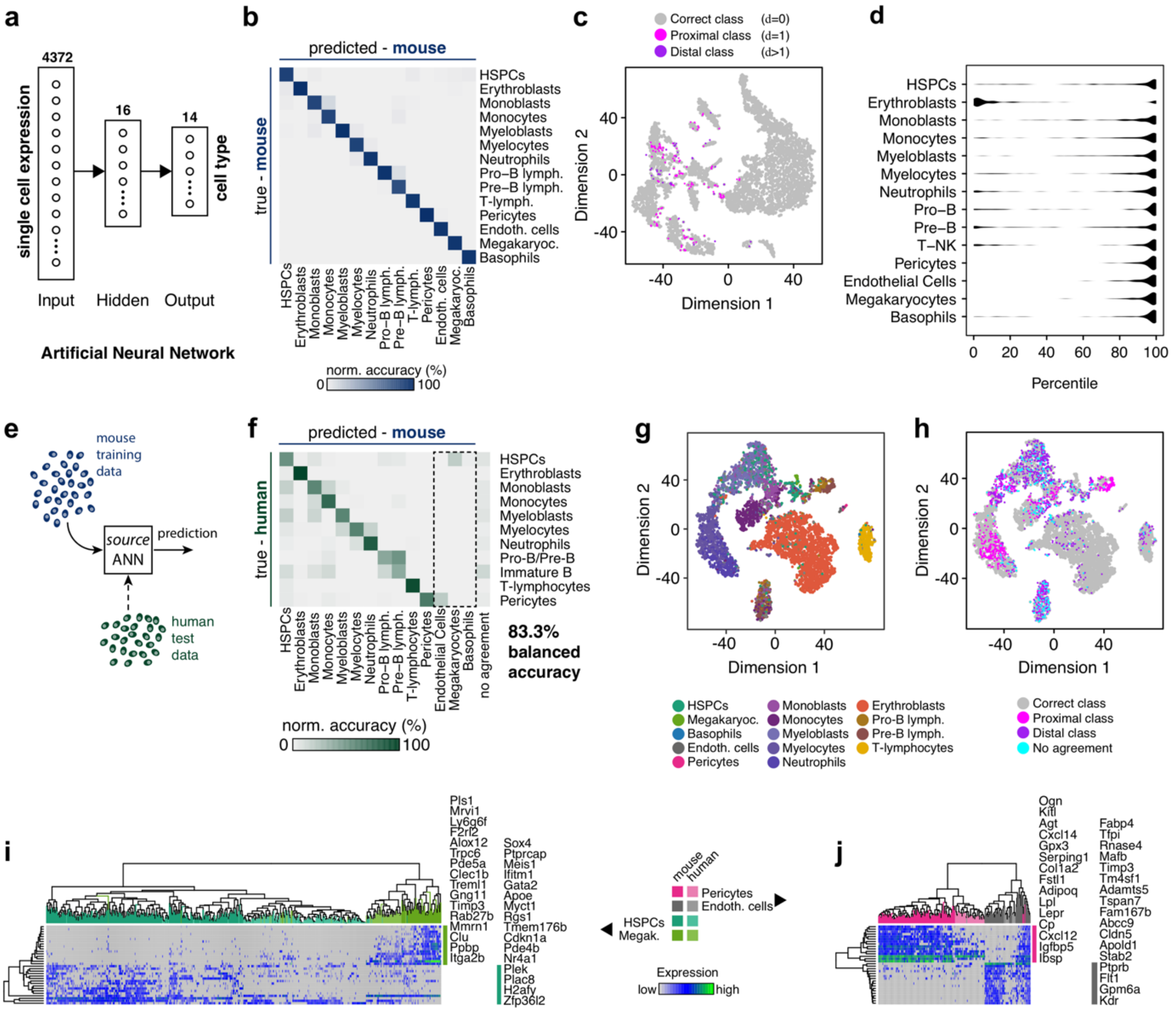

Supplementary Figure 3. Related to Figure 2. (a) Schematic of the artificial neural network (ANN) model used to identify cell types from gene expression profiles obtained from mouse bone marrow cell samples. The ANN consists of an input layer consisting of 4372 units, a 16-unit hidden layer and a 14-class SoftMax output layer (see **Methods** for further details). **(b)** Confusion matrix of validation data, showing accurate classification of cell identities by the ANN. Data displayed is the average over 5-fold cross-validation. **(c)** Distribution of misclassified cells in the training data. Color represents the distance *d* between the true and predicted label in the cell lineage tree in **Fig. 1d**. Cells for which the 5 classifiers did not agree are shown in turquoise. (**d**) Important features from sensitivity analysis of the ANN classifier intersect with the most important features of the MLR classifier. For the top 100 ANN features from **Supplementary Data Table 3**, the distribution of percentile ranks from MLR sensitivity analysis is shown. Since weights can be positive or negative a high or low value indicates that a given feature was identified by both the ANN and the MLR as important. Accumulation of values around 0 and 100% therefore indicates concordant assessment of feature importances by the ANN and MLR. **(e)** Schematic of the transfer process. ANN trained in the source domain (mouse) is used to classify test data from the target domain (human). **(f)** Confusion matrix of classification consensus from 5-fold cross validation. The dashed box highlights cell types identified in the mouse but not the human data. **(g-h)** Projection of human data onto two dimensions using tSNE. Points represent cells coloured by (g) predicted cell identity or (h) misclassification. **(i-j)** Co-clustering of expression patterns in mouse and human cells discriminates human HSPCs from megakaryocytes (i) and pericytes from endothelial cells (j). In both panels clustering is performed using the top-ranking genes from sensitivity analysis.

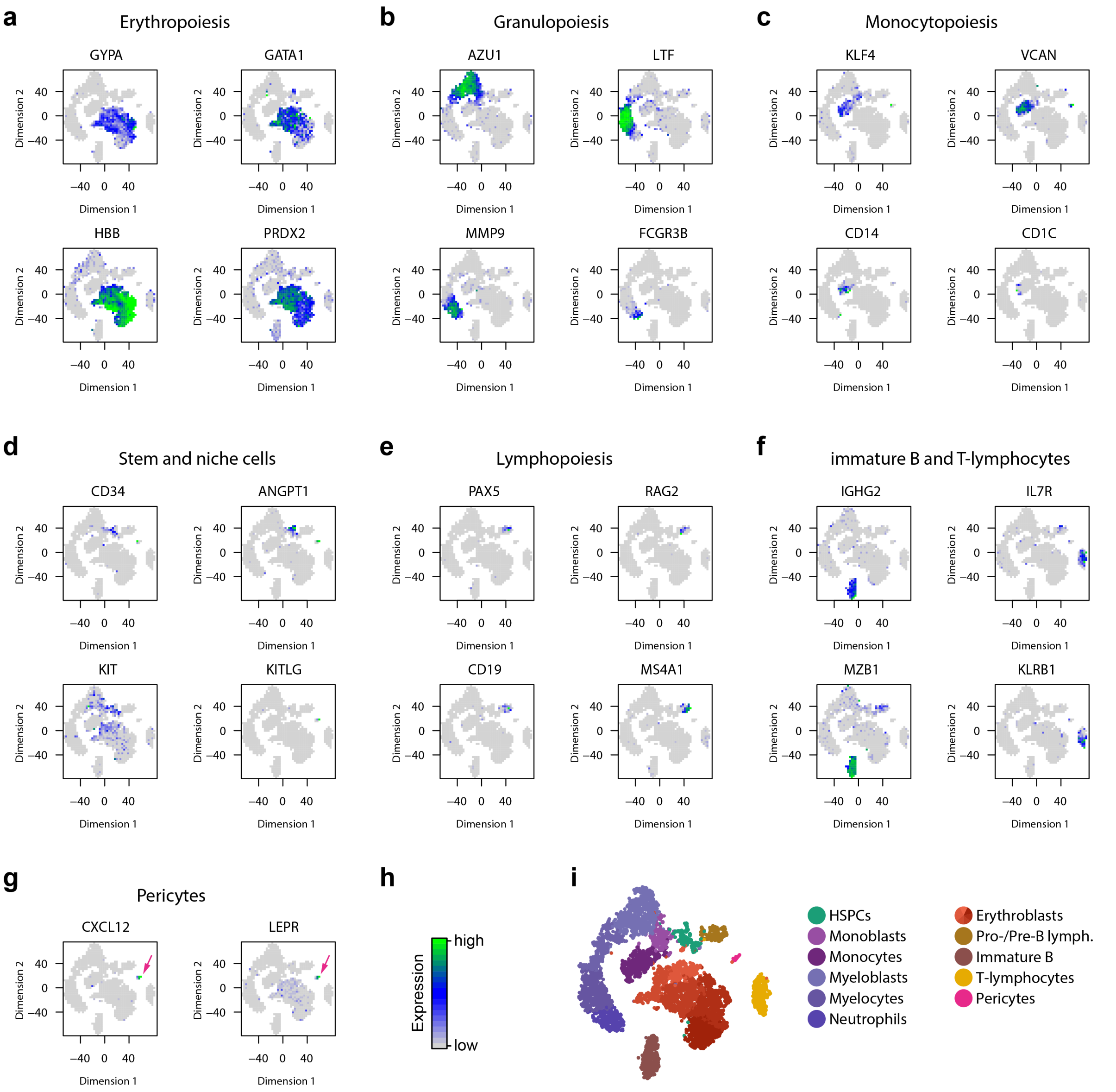

Supplementary Figure 4. Gene expression localizes to developmental branches of human hematopoiesis. a-g Average gene expression superimposed onto 2D embedding of scRNAseq data using tSNE. Localized expression indicative of **a** erythropoiesis, **b** granulopoiesis, **c** monocytopoiesis, **d** hematopoietic stem and progenitor cells and niche cells, **e** endothelial cells and pericytes, **f** lymphopoiesis, **g** pericytes. **h** Color scale. **i** Unsupervised clustering and annotation derived from the literature.

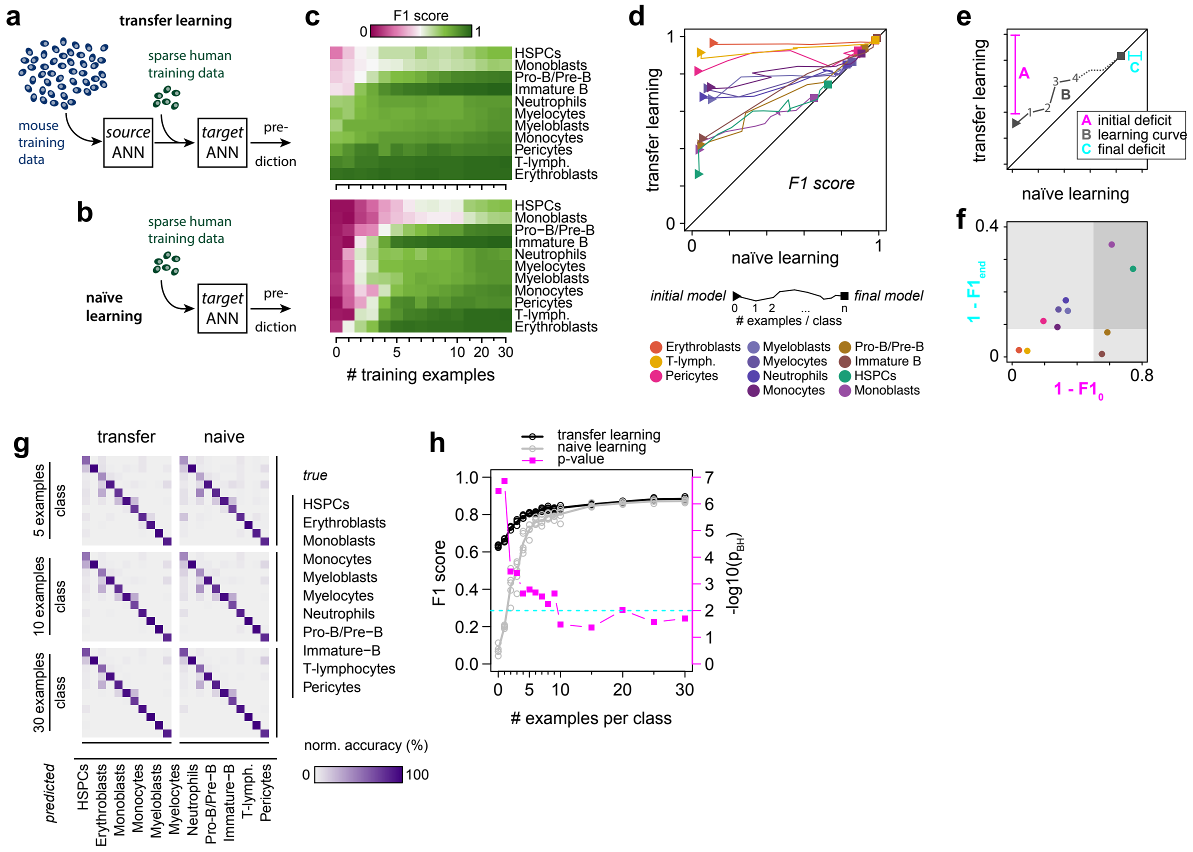

Supplementary Figure 5. Related to Fig. 3. Comparison of transfer learning and naïve learning using the artificial neural network (**ANN**). **(a)** Schematic of the transfer learning process in which a pre-trained ANN is fine-tuned using limited additional data. **(b)** Schematic of the naïve learning process in which an ANN is trained from random initial conditions using limited human data. **(c)** Heatmap of F1 score given training with 0, 1, … 10, 15, 20, 25, 30 examples per class. **(d)** Comparison of F1 scores as a function of the number of training examples (the characteristic learning curves), obtained through transfer learning or naïve learning. **(e)** Schematic illustrating the initial learning deficit ($1 - F_0$) and the final learning deficit ($1 - F_{end}$), indicating how well the model trained in the source domain performs in the target domain (initial deficit; low values indicates good direct transfer), and how well the model from the source domain has adapted to the target domain following fine-tuning (final deficit; low values indicate good adaptation). **(f)** Scatterplot of initial and final learning deficits indicates three four groups of cell types (see main text for an interpretation of this grouping). **(g)** Confusion matrices at various levels of re-training (5, 10, 30 examples per class) for transfer learning and naïve learning. Displayed is the normalized accuracy (number of predicted positives divided by the number of true positives). **(h)** Average F1 score over all classes from 5-fold cross validation (primary *y*-axis) for transfer learning (black) and naïve learning (grey). Solid lines mark the average from cross validation. Negative logarithm (base 10) of *p*-values (FDR corrected; secondary *y*-axis) from one-tailed paired t-tests (alternative hypothesis: $F1_{transfer,\,n} > F1_{naive,\,n}$, for n = 0,1, … 10, 15, 20, 25, 30 examples per class, n=5 from 5-fold cross validation). Dashed line denotes a significance level of $\alpha = 0.01$.

AS scores different:

AS_{max}	AS_{low}	Condition
Coding	Coding	Only if GE tag the same
Coding	Non-coding	
Non-coding	Coding	
Non-coding	Non-coding	

AS scores identical:

$AS_{max\ 1}$	$AS_{max\ 2}$	Condition
Coding	Coding	Only if GE tag the same
Coding	Non-coding	
Non-coding	Coding	
Non-coding	Non-coding	

Supplementary Figure 6. Dual mapped reads.

Green colour indicates alignment to be altered, yellow colour indicates if condition is met.

1 max AS score:

AS_{max}	$AS_{low\ 1}$	$AS_{low\ 2}$	Condition
Coding	Coding	Coding	Only if GE tags the same
Coding	Coding	Non-coding	Only if GE tags the same
Coding	Non-coding	Coding	Only if GE tags the same
Coding	Non-coding	Non-coding	
Non-coding	Coding	Non-coding	
Non-coding	Non-coding	Coding	
Non-coding	Coding	Coding	
Non-coding	Non-coding	Non-coding	

2 max AS scores:

$AS_{max\ 1}$	$AS_{max\ 2}$	AS_{low}	Condition
Coding	Coding	Coding	Only if GE tags the same
Coding	Coding	Non-coding	Only if GE tags the same
Coding	Non-coding	Coding	Only if GE tags the same
Non-coding	Coding	Coding	Only if GE tags the same
Coding	Non-coding	Non-coding	
Non-coding	Coding	Non-coding	
Non-coding	Non-coding	Non-coding	

3 max AS scores:

$AS_{max\ 1}$	$AS_{max\ 2}$	$AS_{max\ 3}$	Condition
Coding	Coding	Coding	Only if GE tags the same
Coding	Coding	Non-coding	Only if GE tags the same
Coding	Non-coding	Coding	Only if GE tags the same
Non-coding	Coding	Coding	Only if GE tags the same
Coding	Non-coding	Non-coding	
Non-coding	Coding	Non-coding	
Non-coding	Non-coding	Coding	
Non-coding	Non-coding	Non-coding	

Supplementary Figure 7. Triple mapped reads.

Green colour indicates alignment to be altered, yellow colour indicates if condition is met.

1 max AS score:

AS_{max}	$AS_{low\ 1}$	$AS_{low\ 2}$	$AS_{low\ 3}$	Condition
Coding	Coding	Coding	Coding	Only if GE tags the same
Coding	Coding	Coding	Non-coding	Only if GE tags the same
Coding	Coding	Non-coding	Coding	Only if GE tags the same
Coding	Non-coding	Non-coding	Coding	Only if GE tags the same
Non-coding	Any combination of Coding / Non-coding			

2 max AS scores:

$AS_{max\ 1}$	$AS_{max\ 2}$	$AS_{low\ 1}$	$AS_{low\ 2}$	Condition
Coding	Coding	Any combination of Coding / Non-coding		Only if GE tags the same
Coding	Non-coding	Any combination of Coding / Non-coding		Only if GE tags the same
Non-coding	Coding	Any combination of Coding / Non-coding		Only if GE tags the same
Non-coding	Non-coding	Any combination of Coding / Non-coding		

3 max AS scores:

$AS_{max\ 1}$	$AS_{max\ 2}$	$AS_{max\ 3}$	AS_{low}	Condition
Coding	Coding	Coding	Coding / Non-coding	Only if GE tags the same
Coding	Coding	Non-coding	Coding / Non-coding	Only if GE tags the same
Coding	Non-coding	Coding	Coding / Non-coding	Only if GE tags the same
Non-coding	Coding	Coding	Coding / Non-coding	Only if GE tags the same
Coding	Non-coding	Non-coding	Coding / Non-coding	Only if GE tags the same
Non-coding	Coding	Non-coding	Coding / Non-coding	Only if GE tags the same
Non-coding	Non-coding	Coding	Coding / Non-coding	Only if GE tags the same
Non-coding	Non-coding	Non-coding	Coding / Non-coding	

All 4 AS scores the same:

$AS_{max\ 1}$	$AS_{max\ 2}$	$AS_{max\ 3}$	$AS_{max\ 4}$	Condition
Coding	Coding	Coding	Coding	Only if GE tags the same
Coding	Coding	Coding	Non-coding	Only if GE tags the same
Coding	Coding	Non-coding	Coding	Only if GE tags the same
Coding	Non-coding	Coding	Coding	Only if GE tags the same
Coding	Coding	Non-coding	Non-coding	Only if GE tags the same
Coding	Non-coding	Non-coding	Coding	Only if GE tags the same
Non-coding	Non-coding	Coding	Coding	Only if GE tags the same
Coding	Non-coding	Non-coding	Non-coding	
Non-coding	Coding	Non-coding	Non-coding	
Non-coding	Non-coding	Coding	Non-coding	
Non-coding	Non-coding	Non-coding	Coding	
Non-coding	Non-coding	Non-coding	Non-coding	

Supplementary Figure 8. Quadruple mapped reads.

Green colour indicates alignment to be altered, yellow colour indicates if condition is met.
