# Supplementary material for: Transfer learning efficiently maps bone marrow cell types from mouse to human using single-cell RNA sequencing
Source: Commun Biol. 2020 Dec 4;3:736. doi: 10.1038/s42003-020-01463-6 (PMC7718277; doi:10.1038/s42003-020-01463-6)
Supplement: Supplementary file 2 — Description of Additional Supplementary Files [file 42003_2020_1463_MOESM2_ESM.pdf]

Description of Additional Supplementary Files

File Name: Supplementary Data 1 - 4 (zip file)

Description: This .zip file contains the following Supplementary Data files:

Supplementary Data 1: Ranked list containing the top 100 features associated with each cell type, from sensitivity analysis of the multiclass logistic regression

Supplementary Data 2: Ranked list containing the top 100 features associated with each cell type, from sensitivity analysis of the artificial neural network

Supplementary Data 3: Results from Gene Ontology analysis of features listed in Supplementary Data 2

Supplementary Data 4: Results from Gene Ontology analysis of features listed in Supplementary Data 1
