# Supplementary material for: Transfer learning efficiently maps bone marrow cell types from mouse to human using single-cell RNA sequencing
Source: Commun Biol. 2020 Dec 4;3:736. doi: 10.1038/s42003-020-01463-6 (PMC7718277; doi:10.1038/s42003-020-01463-6)
Supplement: Supplementary file 5 — Reporting Summary [file 42003_2020_1463_MOESM5_ESM.pdf]

nature research

Corresponding author(s): Patrick Stumpf, Fumio Arai, Ben MacArthur

Last updated by author(s): 13/07/20

Reporting Summary

Nature Research wishes to improve the reproducibility of the work that we publish. This form provides structure for consistency and transparency in reporting. For further information on Nature Research policies, see Authors & Referees and the Editorial Policy Checklist .

Statistics

For all statistical analyses, confirm that the following items are present in the figure legend, table legend, main text, or Methods section.

n/a	Confirmed	
☐	☒	The exact sample size (*n*) for each experimental group/condition, given as a discrete number and unit of measurement
☐	☒	A statement on whether measurements were taken from distinct samples or whether the same sample was measured repeatedly
☐	☒	The statistical test(s) used AND whether they are one- or two-sided<br>*Only common tests should be described solely by name; describe more complex techniques in the Methods section.*
☐	☒	A description of all covariates tested
☐	☒	A description of any assumptions or corrections, such as tests of normality and adjustment for multiple comparisons
☐	☒	A full description of the statistical parameters including central tendency (e.g. means) or other basic estimates (e.g. regression coefficient) AND variation (e.g. standard deviation) or associated estimates of uncertainty (e.g. confidence intervals)
☐	☒	For null hypothesis testing, the test statistic (e.g. *F*, *t*, *r*) with confidence intervals, effect sizes, degrees of freedom and *P* value noted<br>*Give P values as exact values whenever suitable.*
☒	☐	For Bayesian analysis, information on the choice of priors and Markov chain Monte Carlo settings
☒	☐	For hierarchical and complex designs, identification of the appropriate level for tests and full reporting of outcomes
☒	☐	Estimates of effect sizes (e.g. Cohen's *d*, Pearson's *r*), indicating how they were calculated

Our web collection on statistics for biologists contains articles on many of the points above.

Software and code

Policy information about availability of computer code

Data collection	NextSeq System Suite (Illumina, v2.0 and higher); bcl2fastq (Illumina, v1.8.4); STAR (version 2.5.2b); DropSeq tools (v1.0; Macosko et al. 2015)
Data analysis	R (v3.5.0), Seurat package (v2.3.1), Keras package (v2.2.4) with TensorFlow backend (v1.8.0; https://www.tensorflow.org/), DAVID (v6.8; https://david.ncifcrf.gov/).

For manuscripts utilizing custom algorithms or software that are central to the research but not yet described in published literature, software must be made available to editors/reviewers. We strongly encourage code deposition in a community repository (e.g. GitHub). See the Nature Research guidelines for submitting code & software for further information.

Data

Policy information about availability of data

All manuscripts must include a data availability statement. This statement should provide the following information, where applicable:

- Accession codes, unique identifiers, or web links for publicly available datasets
- A list of figures that have associated raw data
- A description of any restrictions on data availability

Data reported in this work are available from ArrayExpress under accession E-MTAB-8629 and E-MTAB- 8630.

Field-specific reporting

Please select the one below that is the best fit for your research. If you are not sure, read the appropriate sections before making your selection.

☒ Life sciences ☐ Behavioural & social sciences ☐ Ecological, evolutionary & environmental sciences

Life sciences study design

All studies must disclose on these points even when the disclosure is negative.

Sample size	n/a
Data exclusions	Unscaled data was discretized (threshold >0) and the union of genes from both species previously identified as variable (threshold for mean > 0.0125, and mean < 4; and log of dispersion > 0.5) in Seurat were selected for training if they were unambiguous orthologues.
Replication	Samples were collected from three independent animals or human research participants.
Randomization	No experimental groups were defined.
Blinding	n/a

Reporting for specific materials, systems and methods

We require information from authors about some types of materials, experimental systems and methods used in many studies. Here, indicate whether each material, system or method listed is relevant to your study. If you are not sure if a list item applies to your research, read the appropriate section before selecting a response.

Materials & experimental systems

n/a	Involved in the study	
☐	☒	Antibodies
☒	☐	Eukaryotic cell lines
☒	☐	Palaeontology
☐	☒	Animals and other organisms
☐	☒	Human research participants
☒	☐	Clinical data

Methods

n/a	Involved in the study	
☒	☐	ChIP-seq
☒	☐	Flow cytometry
☒	☐	MRI-based neuroimaging

Antibodies

Antibodies used	CD45 (Miltenyi Biotec, 130-045-801), CD235a (Miltenyi Biotec, 130-050-501), CD45 (Miltenyi Biotec, 130-052-301), TER119 (Miltenyi Biotec, 130-049-901).
Validation	See manufacturer's website.

Animals and other organisms

Policy information about studies involving animals; ARRIVE guidelines recommended for reporting animal research

Laboratory animals	female, 8-week old C57BL/6 mice
Wild animals	n/a
Field-collected samples	n/a
Ethics oversight	All experimental work including mice were approved by the Kyushu University animal experiment committee.

Note that full information on the approval of the study protocol must also be provided in the manuscript.

Human research participants

Policy information about studies involving human research participants

Population characteristics	Age: 54, 77, 88. Gender: female.
Recruitment	Patients undergoing routine hip replacement surgery were recruited for this study. Informed consent was obtained from these patients.
Ethics oversight	Use of human tissue was approved by the Southampton regional ethics committee (reference 18/NW/0231).

Note that full information on the approval of the study protocol must also be provided in the manuscript.
